# Review of Existing Knowledge and Practices of Tarping for the Control of Invasive Knotweeds

**DOI:** 10.3390/plants10102152

**Published:** 2021-10-11

**Authors:** Marie-Anne Dusz, François-Marie Martin, Fanny Dommanget, Anne Petit, Caroline Dechaume-Moncharmont, André Evette

**Affiliations:** 1 LESSEM, INRAE, Université Grenoble Alpes, 2 rue de la Papeterie-BP 76, 38402 St-Martin-d’Hères, France; fanny.dommanget@inrae.fr (F.D.); andre.evette@inrae.fr (A.E.); 2 Laboratoire Cogitamus, 38000 Grenoble, France; francois-marie.martin@inrae.fr; 3 SNCF Réseau, 6 avenue François Mitterrand, 93574 La Plaine Saint Denis, France; anne.petit@reseau.sncf.fr; 4 SNCF Réseau, 19 avenue Georges Pompidou, 69003 Lyon, France; caroline.dechaume@reseau.sncf.fr

**Keywords:** ground covering, *Reynoutria*, *Fallopia*, *Polygonum*, knotweed management, geotextile, invasive alien plants

## Abstract

Managing invasive exotic plant species is a complex challenge, especially for Asian knotweeds (*Reynoutria* spp.). Tarping is a regularly cited but poorly documented control method, which consists of covering the ground with a tarp (agricultural tarp, geotextile, geomembrane, etc.) to create a physical barrier to hinder plant growth and deprive the plants of light in order to deplete their rhizomatous reserves. To improve our knowledge of tarping in order to identify the key factors of its success or failure, we reviewed the relevant grey and scientific literature and conducted an international survey among managers to collect feedback on tarping experiments. In the literature, as well as in the field, practices are quite heterogeneous, and the method’s effectiveness is highly contrasted. A better consideration of knotweed biology may improve the efficacy of the method. Based on the bibliography and survey work, we propose practical recommendations including covering the entire stand, extending the tarping up to 2.5 m beyond its edges for a period of at least six years, and ensuring regular monitoring. Even though tarping does not seem to be a one-size-fits-all solution to eradicate knotweed, it could still be a useful control method once knotweed has become a critical management issue.

## 1. Introduction

The number of invasive alien plant species (IAS) is increasing worldwide mainly as a result of human activities [1]. These species are causing various detrimental impacts on biodiversity, ecosystem services, and human health and activities [2], to such an extent that the Invasive Species Specialist Group (ISSG) of the UICN and the Intergovernmental Science-Policy Platform on Biodiversity and Ecosystem Services (IPBES) consider them one of the main threats to biodiversity at the global level [3,4]. As a consequence, invasive species have become a global concern. Managing invasives and the damage they cause is extremely costly: the annual mean cost at the global scale has been estimated at US$26 billion over the past few decades, and may have reached US$162.7 billion in 2017 according to one study [5]; total IAS-related costs are estimated to be at least €12.5 billion per year in the European Union [6], and €38 million in France alone [7]. Therefore, many countries have adopted policies and strategies to deal with biological invasions. The Convention on Biological Diversity took invasive species into account at the international level in 2011 (Aichi Biodiversity Target 9) and the European Union adopted a regulation (EU No 1143/2014) on the prevention and management of the introduction and spread of IASs in 2014.

In the field, IAS control is a complex challenge for land managers (such as transport infrastructure or natural area managers, hereafter referred to as managers). They have to design local-scale strategies with often limited financial and human resources [8,9] and without being certain of the best control options to implement [10,11]. While IAS management literature is extensive, there is still a “knowing-doing gap” (sensu Matzek et al. [12], i.e., a gap between research and practice), as scientific research rarely meets the manager’s needs [13,14]. The complexity of invasion processes and the diversity of species involved also make it difficult to develop effective generalized control methods [15]. For many species, managers often face an absence of consensus [10] and even sometimes contradictory management recommendations [16]. They may also face division in the scientific community concerning IAS impacts and the usefulness of costly management programs [17]. In addition, climate change will likely challenge effective IAS management practices [9]. In such a context of uncertainty [18], management choices rely heavily on local managers’ own perception of the IAS, their personal experiences, and advice from fellow managers, more than on the most recent scientific knowledge [19].

Japanese knotweed s.l figures among the world’s 100 worst invasive alien species [20] and, as such, is a good example of the challenges faced by scientists and managers. The species complex is composed of the Japanese knotweed (*Reynoutria japonica* Houtt.; syn. *Fallopia japonica* (Houtt.) Ronse Decr. or *Polygonum cuspidatum* Siebold & Zucc.), the Giant knotweed (*Reynoutria sachalinensis* (F. Schmidt) Nakai; syn. *Fallopia sachalinensis* (F. Schmidt) Ronse Decr. or *Polygonum sachalinense* F. Schmidt), and the hybrid of the two: the Bohemian knotweed (*Reynoutria* × *bohemica* Chrtek & Chrtková; syn. *Fallopia* × *bohemica* (Chrtek & Chrtková) J. P. Bailey or *Polygonum* × *bohemicum* (Chrtek & Chrtková) P. F. Zika et al. Jacobson) [21]. Japanese and Giant knotweeds originated from Eastern Asia and were introduced into Europe in the 19th century, mainly as ornamental plants. These invasive perennial geophytes [22] are now widely naturalized in Europe, North America, Australia, New Zealand, Chile, and South Africa [23], where they form large monocultures, called “stands” or “patches”, in a wide variety of habitats. Riverbanks, roads, and railways, as well as wastelands and manmade habitats are particularly concerned since the plants spread primarily through the dispersal of vegetative propagules (e.g., fragments of stem or rhizome) and this is facilitated by earth-moving activities and floods.

Though still under discussion [24,25], the numerous negative impacts of knotweeds [26,27,28,29] and their locally high rate of spread are forcing managers to take action. A growing body of literature proposes many different control methods including cutting, mowing, pulling, digging, tarping, burning, grazing, planting, salting, and using herbicides or biological control [30,31,32,33]. While recommendations for all of these techniques may be found in various management practice guides, their effectiveness has rarely been scientifically assessed in readily available, long-term, robust, controlled experiments [34]. Furthermore, to date, no manual, mechanical, or chemical method has been recognized as fully effective against knotweeds, or fully compatible with sustainable management goals [10,31,34,35]. Worse still, some control techniques could even increase the mean lateral spread of knotweed stands [36,37] or favor the dispersal of knotweed propagules [23,38]. While considerable effort and huge amounts of money are being invested, the lack of information and even consensus on the best knotweed control methods does not allow managers to confidently predict the success of their management campaigns [10].

**Figure 1:**
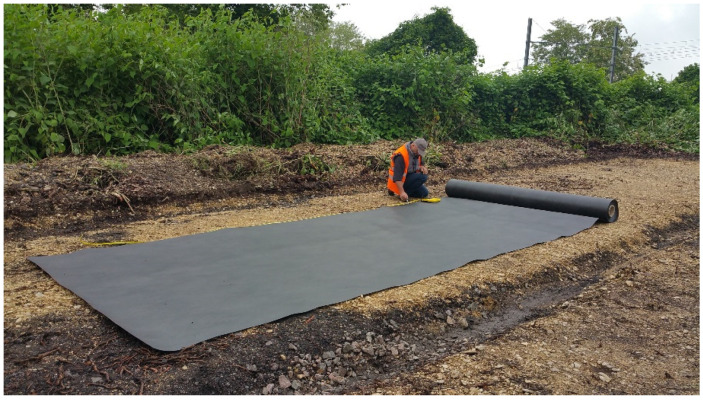
Installation of a synthetic geotextile as part of a tarping operation to control Asian knotweed. © INRAE.

One technique that is regularly cited in the literature, despite little rigorous documentation, is “tarping”. A mechanical method, tarping consists of opposing a physical barrier to plant growth over a period of time (Figure 1). This method is designed to deprive the knotweed of light, thus preventing photosynthesis, until complete depletion of the reserves stored in their belowground organs. The rhizomatous system of knotweeds, which may account for two-thirds of the plant’s total biomass, allows them to store and share considerable amounts of resources among shoots to maximize the growth of the whole stand and guarantee its resilience in case of disturbances [26,39,40]. The knotweeds’ rhizomatous systems can extend 3 m down into the soil. They can expand laterally from aboveground stems over several meters, with most typically less than 2.5 m and rarely up to 4 m [41].

Tarping is also frequently used in production agriculture and by home gardeners as a soil solarization technique. During solarization, the soil covered with a plastic tarp is heated by solar radiation and reaches a temperature high enough to kill weeds and other plant pests [42]. The effects of solarization on weeds is species dependent, as time and temperature requirements for thermal death may vary considerably among weed species [43]. The method’s effectiveness is determined by soil moisture, climate, aspect, and weather but also by the different materials used to cover the soil [44]. Various materials, from the simplest to the most sophisticated, are used for knotweed tarping, including geotextiles (permeable fabrics), agricultural tarps, and geomembranes (i.e., waterproof fabrics), either synthetic or biodegradable. More recently, tarps specifically designed to prevent invasive plant growth have also been developed [45].

As with other control methods, robust evidence is lacking to assess the effectiveness of tarping to control knotweed populations. The few documented studies suggest mixed results [46]. To help fill this knowledge gap and favor well-informed management, the practice of tarping needs to be thoroughly investigated. We therefore propose an overview of tarping practices in order to identify some likely factors for the success or failure of the method: (i) we reviewed the scientific and technical literature related to knotweed tarping, and (ii) we conducted an international survey addressed to managers in French- and English-speaking countries where invasive knotweeds are present.

## 2. Materials and Methods

### 2.1. Bibliographic Review

As we were interested in both grey and scientific literature, in spring 2020, we searched for references on Google Scholar and the standard Google search engine with the following keywords in French: ‘bâchage’ or ‘géotextile’ or ‘solarisation’ or ‘gestion’ AND ‘renouée’ or ‘Fallopia’ or ‘Reynoutria’ or ‘Polygonum’; and in English: ‘tarping’ or ‘covering’ or ‘sheeting’ or ‘geotextile’ or ‘solarization’ or ‘management’ AND ‘Japanese knotweed’ or ‘Fallopia’ or ‘Reynoutria’ or ‘Polygonum’. We screened each French or English document for relevance. As our focus was on pure in situ tarping, we deliberately chose to exclude ex situ tarping operations (i.e., when knotweed rhizomes are extracted and then tarped on another site) and operations where the soil is crushed with a stone crusher prior to tarping, a technique developed in France (cf. [47]). We read each selected document attentively in order to summarize the available relevant information on the method. Particular attention was paid to the objectives pursued, the environments of use, the protocols followed, and the efficacy described by the authors in order to identify the key factors influencing tarping success or failure.

### 2.2. Survey of Knotweed Control through Tarping

To complete the existing published knowledge on knotweed tarping techniques, we collected feedback on unpublished field trials via an online questionnaire. The questionnaire, in both French and English (Supplementary File S1), was available online from May to July 2020 and was sent to various manager mailing lists in French- and English-speaking countries where invasive Asian knotweeds are present. We targeted managers who already had experience with the tarping method. The survey was divided into nine sections: general information regarding the tarping project, site description, site preparation before tarping, tarping installation, monitoring operations, tarp removal, post-control observations, cost, and the manager’s perception. Presumed key technical points were addressed through specific questions. As such, our questionnaire mostly contained check-all-that-apply questions, rating scale questions, and some open-ended response questions. Most questions included an “other” option in order to collect unexpected answers. Since processing the free responses would have taken a significant amount of time and, as there are few documented tarping experiments, we decided to focus on a descriptive analysis of the data.

## 3. Results

### 3.1. Results of the Bibliographic Review

We found 63 relevant documents referring to tarping as a control method for invasive knotweeds (Supplementary File S2). The oldest was published in 2004. However, we only found three references that quantitatively assessed the effectiveness of tarping in comparison to other control methods [31,48,49]. Most of the collected documents were Best Management Practice Guides, technical reports, and conference proceedings. We also found numerous references to tarping operations in local newspapers and internet reports, but we did not include them in an exhaustive list as they often lacked rigor. Our review confirmed both stakeholders’ and managers’ interest in the method, despite the lack of scientific evaluation of its effectiveness, and many of the documents attempt to provide a methodology and guidance for the proper use of tarping (see Table 1).

#### 3.1.1. Management Objectives and Environmental Context of Tarping Application

The literature review showed that tarping has been used on knotweeds to achieve different objectives. The most common objective is to create a physical barrier to plant growth and to prevent photosynthesis [50,51,52,53,54,55]. Regrowth (i.e., regenerating shoots) is blocked by the fabric, thus depleting the carbohydrate reserves stored in the rhizomes. A solarization effect is also frequently sought according to the literature. In this case, the fabric acts as a medium designed to expose knotweeds to high temperatures [55] that could “cook the root system” [50,52]. More rarely, tarping has been used to deprive the plant of water by covering it with an impermeable membrane, i.e., a geomembrane (Mireille Boyer, pers. comm.). These objectives, depending on the authors, can be pursued separately or in a complementary approach. In any case, the overall goal is to weaken the rhizomes as much as possible [32,50,53,55,56,57].

Typically, tarping is only recommended in specific environments or contexts, and for small knotweed stands, but recommendations vary according to country and author. The method is particularly recommended when the use of herbicides is prohibited [58] and is sometimes only considered in that case [32]. This is particularly noticeable in the USA. For example, we found about 10 cases of tarping in the State of Washington [56,59,60,61] that started in the 2000s after herbicide bans. However, these tarping operations stopped and were replaced by chemical treatments as early as 2010 after a change in the law reauthorized the use of herbicides [61]. In the Cedar River municipal watershed, tarping seems to be currently used only as a complement in case of regrowth on areas already treated with herbicides at least three times [61]. Tarping in the UK is a special case. Knotweed is very prevalent throughout the country and can detrimentally affect the price of real estate [41]. Tarping is mainly used in areas where building construction is planned in order to protect structures and hard surfaces: infested areas are sealed horizontally with a tarp before construction or a tarp is laid under the foundations to prevent the knotweed from entering the building [62]. Regarding the use of tarping on riverbanks, there appears to be no consensus among authors. For some, tarping is not suitable for steep or flood-prone sites [63]. Floods can accelerate the degradation of the tarp [48,64], tear off whole tarps or pieces, and disperse them downstream [45]. For others, the tarping method can be applied on a riverbank if suitable fixations are used [51] and if fabrics do not contain leachable chemicals that could pollute watercourses [62]. The method does seem interesting as it prevents stem or rhizome fragments from spreading downstream [53]. According to SPIGEST [55], tarping should be preferred in hard-to-reach and/or sloping areas, where it is difficult to intervene frequently with machinery or to graze animals. However, Guerin and Hedont [53] advise against its use on sites with an aesthetic vocation, where soil life is rich (because of the risks of soil sterilization) or where the site contains too many obstacles. There is a consensus that tarps should be used only on small knotweed stands, although the definition of a small stand varies among authors: “from 50 stems or less” [57] to less than 500 m^2^ [55]. The complexity of tarp installation [53] and the cost of the fabrics [46] do not advocate for the use of this method on large surfaces. According to Soll [57], tarping use should focus on small populations in open, accessible terrain.

#### 3.1.2. Methodological State of the Art

##### Preparatory Work

The first step in setting up a tarping operation, recommended by all authors, is to clear the area and prepare the ground prior to installing the fabric. This step usually consists of mowing down the knotweed stems. It can be completed with manual or mechanical excavation of the rhizomes [65,66,67], as digging up the rhizome crowns may speed up the control process [68]. Before installing the tarp, it is recommended to clean up the area as much as possible by removing all debris, stones, and shrubby vegetation to prevent the tarp from tearing or ripping [51,53]. Authors also frequently advise flattening the area to facilitate tarp installation [65,69]. A layer of mulch can also be spread over the area to prevent the cut stems from puncturing the tarp [58]. These operations must be carried out with great care to avoid spreading knotweed fragments [70].

Recommendations regarding the appropriate timing of these preparations vary among authors, but the reasons for these choices are not always stated. Anderson [50] and Godmaire and Houbart [52] advocate for an intervention in late spring, as does Cygan [58], who states that at this time of year, the root system is weakened because “the plant has exhausted stored carbohydrates”. Others recommend action at the beginning of the year or in spring [32,55,57,71]; according to Branquart et al. [51], intervening during this period limits the volume of waste to be treated after mowing. Interestingly, even though preparation operations are usually carried out just before the tarp is laid, some may mow or excavate the knotweed stand for several years before laying the tarp in order to weaken the plant as much as possible [53,72].

##### Tarp Installation

The next step is to cover the infested area with one or several strips of tarps. Various types of covering fabric have been used in the literature from cardboard (in an unsuccessful experiment [57]) to the most sophisticated geomembrane [65]. However, geotextiles (typical in landscaping) and agricultural tarps are the most frequently recommended. Sometimes a combination of materials is used: agricultural tarps plus geotextiles to prevent holes [72] or thick cardboard plus heavy black plastic tarps [69]. The choice of the tarp is quite important since the strength and longevity of the fabric is a key factor for success [32]. According to Miller et al. [73], a black tarp is more effective than a clear one because it blocks the sunlight. Two [55] or three layers of tarp [72] are sometimes superimposed to improve resistance and efficiency. The Public Service of Wallonia [51] specifies that it is preferable to use non-woven fabrics with a density above 240 g/m^2^.

Due to knotweed growth dynamics and their capacity for lateral expansion, the tarp must be laid beyond the visible limits of the stand and carefully fixed to the ground. Recommendations regarding the necessary distance beyond the stand edge vary greatly from 1 m [55] to 10 m [69] while some recommendations are quite vague: a “few meters” [52] or “further out” [50]. Once laid, the tarp is fixed to the ground by various means. The tarp can simply be weighed down with heavy materials (rocks, logs, etc.) to prevent the wind from blowing it away and to prevent it from being lifted by the knotweed growth that almost always occurs in the first few years. Staples can also be used, either alone or as a complement to weighing the tarp down, but they may create weak points in the tarp through which the knotweed can sprout [58]. Tarp edges are sometimes buried vertically into a trench up to 1.5 m in depth [52] in order to hold the tarp in place and to create a vertical rhizome barrier. When several tarps are used, it is necessary to solidly bind them together (with glue, tape, staples, and/or heat sealing) to prevent sunlight from penetrating and to provide sufficient overlap (60 cm [58]; 30 cm [51]) so that knotweed shoots cannot grow through. The fabric is sometimes covered with soil or mulch for aesthetic reasons, but this also protects the fabric from UV rays [58] and helps hold the tarp to the ground. Finally, some guidelines recommend planting trees or shrubs of local species at the same time to create competition for the knotweed [74]. However, deliberately piercing the fabric for plantations creates weak points and is sometimes discouraged [51].

#### 3.1.3. Post-Tarping Follow-ups

##### Monitoring

Frequent monitoring of the treated area is usually advised to ensure that the fabric or fixations are in a good state of repair, to remove any knotweed regrowth as the plant will likely continue to grow if it has any access to sunlight or air [50,65], and to stomp down re-growth under the tarp [55,71]. Consequently, monitoring recommendations range from twice a year [51] to as often as every two to four weeks during the growing season [54,71].

##### Tarping Duration and Fabric Removal

**Figure 2:**
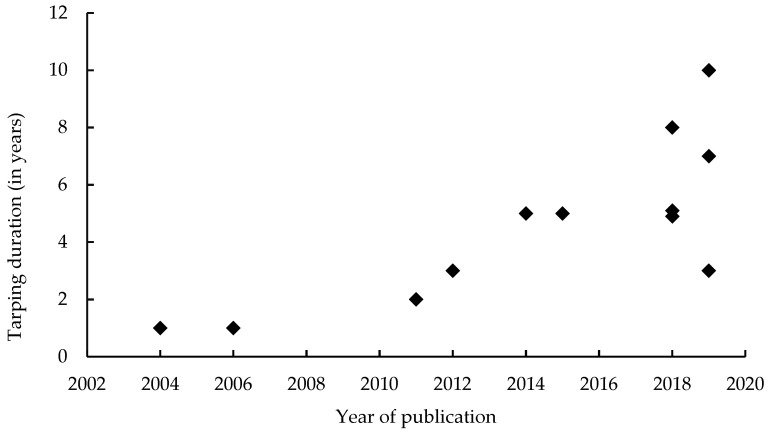
Changes in the recommended tarping duration according to the documents’ publication year.

The literature usually advises removing the tarp after a certain period of time. However, the recommended duration varies greatly from only one growing season [32] to as much as 10 years [53]; the recommended duration seems to be increasing with time (Figure 2). A Norwegian study showed that rhizomes can survive for more than three years under tarps [49] and the current tendency is towards a longer tarping duration. The duration should also be adapted to the characteristics of the area or of the knotweed stand. For example, managers from King County recommend a longer duration if the soil is wet or the population large and well-established [71]. To ensure that the tarp is removed at the right time, it may be useful to open a test window in the tarp for at least one season to ensure that the knotweed has been eradicated before removing the fabric [53]. Although most authors advise removing the tarps [32,57], especially if they are made of synthetic or plastic materials [75], others do not, particularly when the tarp has been covered with soil or mulch [51].

#### 3.1.4. Tarping Effectiveness

The effectiveness of the method is assessed differently depending on the authors. Some consider tarping to be a very effective alternative to herbicides [58], particularly suitable for the control of woody or clonal herbaceous plants [53]. In other (older) publications, there were no reports of successful long-term control with tarping alone [32,57]. Several operations in the USA were considered successful after five or six years of tarping but only on very small patches; on larger patches, despite eight years of continuous tarping, eradication was unsuccessful [56,61]. The scientific studies we reviewed reported mixed results, making it impossible to conclude on tarping effectiveness. While Jones et al. [31] considered geomembrane tarping to be the least effective technique for controlling knotweed (in comparison with herbicides and integrated physiochemical control treatments), Gerber et al. [48] showed that tarping reduced knotweed biomass but advised against using the technique in flooded environments. Kaczmarek-Derda et al. [49] showed that knotweed rhizomatous systems can survive more than three years and that longer tarping results in a substantial reduction of new shoots. Other than direct eradication, the method can also be used as a control technique [72], which, with regular maintenance, may lead to eradication in the long run [51,63], or at least be a first step in depleting the knotweed’s rhizomatous reserves before reinforcing competition by restoring native plant communities [55]. Finally, some consider that the success mostly depends on the rigor with which the method is applied and that the tarping method “is only as good as the way in which the [tarp] has been laid” [62].

The disadvantages and limitations of the technique have also been discussed, for example, the tarp may have potential non-targeted impacts; the habitat value of tarp on the landscape; the bare soil resulting after the tarp’s removal could become a seedbed for other IAS [32,73,76], and the plastic waste from non-biodegradable tarps is difficult to manage [72]; and some authors suggest that the rhizomes may become dormant for several years, rendering the control effort useless [51,52,62,69,77].

### 3.2. Questionnaire Results

We received 81 responses to our questionnaire (Supplementary File S3), 69 of which came from France, while the rest were from the USA (4), Canada (3), Belgium (2), Germany (1), the Netherlands (1), and Slovenia (1).

**Figure 3:**
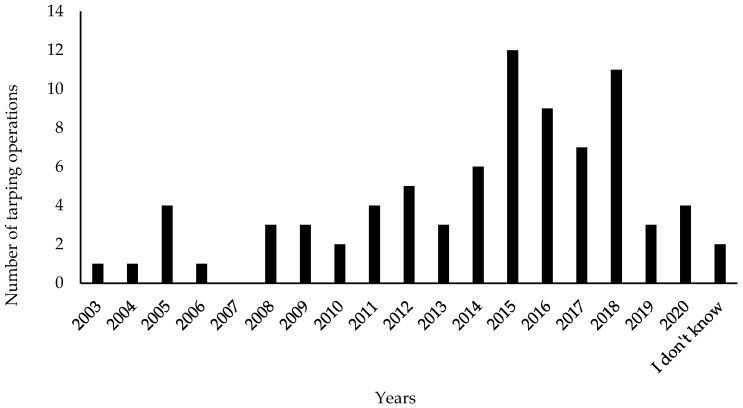
Number of tarping operations identified from the questionnaire according to the starting date of the operation, for a total of 81 tarping operations.

The respondents worked mainly for local authorities (34%) and watershed management organizations (32%). Most respondents felt that they had a good level of knowledge of knotweeds, with a mean score of 6.42 on a scale from 0 (novice) to 10 (expert). The identified tarping operations were launched between 2003 and 2020 (Figure 3). Three quarters of the operations started more than three years ago.

#### 3.2.1. Management Objectives and Environmental Context of Tarping Application

The reported tarping is mainly being used for the sake of comparison with other methods (53%), and most operations are experimental trials. Only 15% of the respondents had had a previously successful experience with tarping. Bibliographical research (38%) and technical or regulatory constraints (42%) also explained the choice of tarping as a control method. Through this method, the managers’ main goal was to eradicate knotweed (69%), to limit knotweed dispersal on the site (54%) and/or to other areas (40%—several answers were possible).

**Figure 4:**
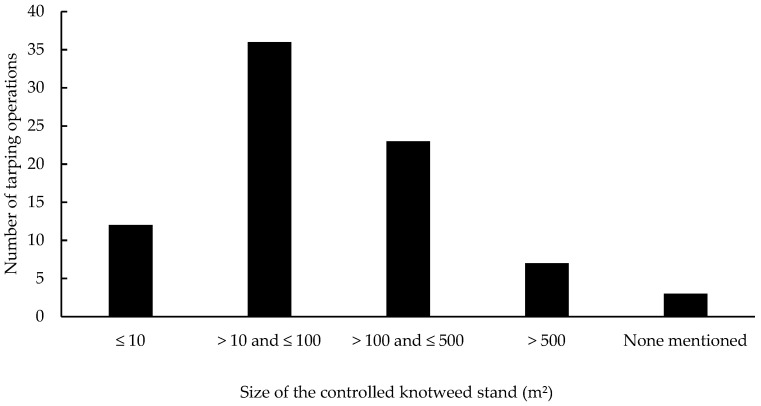
Size of the tarped knotweed stands based on respondents’ answers, for a total of 81 tarping operations.

Tarping was mostly used on riverbanks (47%) and on roadsides (37%). Overall, tarped sites were typically easy to access, sunny, sparsely wooded, and rarely flooded (although 21% were flooded regularly). Operations were apparently set up regardless of slope steepness and focused mainly on small- to medium-sized knotweed stands (Figure 4): half of the tarping trials were carried out on stands < 90 m^2^ and 75% on stands < 230 m^2^ (still, the largest tarped stand covered almost 5000 m^2^).

#### 3.2.2. Methodological State-of-the Art

##### Preparatory Work

In 84% of the cases, managers performed some kind of preparation on the site prior to tarping. Most respondents (56%) applied more than one of the following preparatory measures: mowing (60%), manually or mechanically extracting rhizomes (29%), flattening the area with an excavator (19%), and removing stones (19%). Respondents considered preparation an important step, with an average mark of 7.6 on a scale from 0 (useless) to 10 (essential). Preparation work almost always took place just before the tarp was laid, regardless of the season, since the preparation date was chosen mainly because of organizational constraints (70%). Still, the knotweed biological cycle was also frequently taken into account, as 41% of the respondents favored action before or at the beginning of knotweed emergence in order to limit the amount of waste to treat, 16% wanted to intervene when the rhizomes were the weakest, and 12% intervened before flowering to prevent the formation of seeds.

##### Tarp Installation

Geotextile was the preferred material for tarping operations (43%). Agricultural and other geomembranes were also used (30% and 22%, respectively). Only 10% of the listed operations were carried out with biodegradable geotextiles. In 78% of the cases, the knotweed stands were entirely covered. When the stands were not entirely covered, it was mainly due to technical constraints, though some managers did so on purpose to test the method on a small plot before considering a larger project. Interestingly, 71% of the managers decided to extend the tarped area beyond the initial stand’s edges to cover a buffer zone of at least one meter.

Around three quarters of the projects required the use of several strips of fabric. Various techniques were used to bind the strips together, but staples were most frequently used (50%). On average, the staples were placed every 67 cm (min 6 cm; max 2 m) on strips overlapping by 47 cm on average (min 1 cm; max 1.5 m). Adhesive tape, weighing the fabric down with heavy objects, heat sealing, and glue were also sometimes used, either alone or in conjunction with staples. The tarps were anchored to the ground with staples (52% of the cases, typically a concrete reinforcing bar was pinned down every 71 cm on average) and/or by burying the edges of the sheet in a trench (54%). Fixation was sometimes enhanced by covering the fabric with various materials like gravel or topsoil (37% of the cases, with an average covering layer of 20 cm) or by ballasting with heavy objects (31%). When trenches were dug to fix the edges of the tarp to the ground, they were on average 35 cm deep (with a minimum of 10 cm and a maximum of 1.2 m). The average depth was higher (58 cm with a minimum of 10 cm and a maximum of 2 m) when the objective was also to create a vertical rhizome barrier.

Covered areas usually had few obstacles and 64% of the tarps were installed without any unintentional damage. The few holes identified were mainly due to rocks, rubble, rebar, and branches (45%) present in the ground. Installation was considered moderately difficult, but 80% of the respondents said it was a crucial step in the process. Interestingly, 30% of the respondents carried out planting within the tarped area, either by seeding or by planting cuttings and/or transplants. These managers aimed to create competition between the planted vegetation and the knotweed (67%), to improve the aesthetic aspect of the site (50%) and/or to avoid erosion (38%).

#### 3.2.3. Post Tarping Follow-up

##### Monitoring

After the tarping was installed, the sites were almost always monitored at least once a year (98%), while many managers monitored 3 times a year or more (52%). Monitoring was generally more frequent during the vegetation period in the first few years after installation. While some limited this monitoring to simple visual inspections, most respondents uprooted the knotweed regrowth (66%) and repaired the fabric or fixations (38%).

##### Tarping Duration and Tarp Removal

More than two-thirds (69%) of the respondents planned the tarping duration at the beginning of their project. This planned duration ranged from one to 20 years, with most projects planned to last three years or more (69% of the cases). This duration was based on bibliographical research (35%), peer recommendation (32%), and/or personal experience (21%). For those who did not plan a tarping duration, it was either because they intended to leave the tarp in place or because they wanted to determine the duration according to the results obtained over time. Of all respondents, only 27% had removed their tarps; another 23% expressed their intention to do so when the planned duration had been reached, 33% intended to leave the tarp in place, and finally another 5% said they would leave it because it was too degraded to be removed. Whatever the option planned at the end of the tarping operation, it is mainly synthetic tarps that are used: by 91% of those who had removed their tarps, by 87% of those who expressed their intention to do so, and by 72% of those who intended to leave the tarp in place.

For the operations where the tarps had already been removed (n = 22), in 13 cases, removal was carried out after three years or more, but in seven cases, the managers removed the fabric after only one or two years. Following removal, additional management was performed in 15 cases, usually to restore vegetation cover or to mow down the remaining knotweed shoots.

#### 3.2.4. Tarping Effectiveness

Regrowth of knotweed stems was observed in 78% of the cases, mostly during the first six months after tarp installation (71%). The regrowth appeared mainly within the covered area (54%) and/or in the immediate vicinity (within 2 m of the covered area, 46%). Fragile points in the tarp seem to have been exploited by the knotweed, particularly near obstacles, at holes or where the strips overlapped. The tarping system (the tarps themselves or their anchoring system) deteriorated in 43% of the operations. This degradation was observed shortly after the installation in more than 50% of the cases. Furthermore, 9% of the tarping trials were utterly abandoned.

Of the 22 respondents who proceeded to the removal of the tarp, six said they had successfully eradicated knotweed from the area. Seven managers noted that there were no knotweed plants at the time of the removal, while 10 observed some regrowth under the previously tarped area, and 11 in the immediate vicinity of the previously tarped area.

The 22 respondents who had removed their tarps were able to provide an assessment of the method’s effectiveness on a scale of 0 (ineffective) to 10 (very effective), first at the time of the removal and second, at the time of the managers’ final observation. This final observation was mostly (54%) made within the months or the year following the tarp’s removal. The mean score at the removal was 5.7 (±3.87 standard deviation) and 5.6 (±3.74 standard deviation) at the final observation. Effectiveness ratings at the two dates differed little, though the effectiveness assessment at removal was typically confirmed at final observation. For example, seven respondents deemed the method effective (score of 9 or 10) at the time of removal and nine deemed it effective at their final observation. Conversely, three managers considered the method to be totally ineffective (score of 0 or 1) at the time of removal and five managers gave the lowest score at the time of their final observation.

The respondents considered that tarping was slightly more time-consuming than other knotweed control methods. There was no consensus regarding the cost of tarping, but, on average, they felt that the cost was comparable to other methods. The average cost of tarping in our survey was 34 €/m^2^ though it varied greatly, from 1 €/m^2^ to 167 €/m^2^. However, cost estimation methods differed among managers (monitoring and maintenance were not always included, some added the purchase or hire of equipment needed for installation, others used volunteer labor, etc.), thus partly explaining these differences. However, costs also seem to depend on the type of fabric used. The average cost of an operation with an agricultural tarp (n = 14) was the lowest at 26 €/m^2^, compared to 30 €/m^2^ for an operation with geotextile (n = 19) and 60 €/m^2^ with a geomembrane (n = 9). Finally, 52% of the respondents intended to do new tarping trials at other sites.

### 3.3. Consistency of the Results of the Bibliography and the Questionnaire

#### 3.3.1. Management Objectives and Environmental Context of Tarping Application

The number of responses to our questionnaire and the amount of grey literature found on the subject of tarping shows that the technique is frequently used both in France and abroad, despite a lack of scientific data. Through the results of the questionnaire, we observed an increase in the number of tarping operations over the last few years, suggesting that the method is becoming increasingly popular. The development of more advanced geotextiles specifically dedicated to the control of invasive rhizomatous plants could partially explain this trend. However, these results may also reflect the difficulty of obtaining reliable information about older experiments (e.g., absence of publications, unavailable data).

The method is frequently chosen for comparison with other methods. This confirms that in the ‘war’ against knotweeds, managers are still struggling to find an effective way to eradicate them. While many authors reckon that eradicating knotweed at the large scale is impossible [10,19,78], 69% of the respondents were still interested in locally eradicating knotweed with tarping. Furthermore, eradication is not necessarily the managers’ only goal [79].

Contrary to what is recommended in the literature, managers seem to favor tarping in fairly accessible environments over a wide gradient of slope steepness. Tarping is particularly used on roadsides and riverbanks, even though its use along rivers is controversial in the literature. Theory and practice do agree, however, on the size of the knotweed stands to be controlled. Tarping is mainly practiced on small- to medium-sized stands (75% of the experiments reported through the questionnaire were carried out on stands smaller than 230 m^2^), although we found no detailed justifications for this choice. It is unclear, however, whether managers assume tarping’s effectiveness decreases with increasing knotweed stand size or, as stated by Guerin and Hedon [53] and Lavoie [46], whether the complexity and/or cost of the method prevent it from being applied on larger surfaces.

#### 3.3.2. On Assessing Tarping Effectiveness

It emerges from the review and the questionnaire that properly assessing tarping’s effectiveness is challenging as tarping is implemented in various management and environmental contexts, and the technical characteristics of tarping operations can be extremely diverse (in terms of fabric used, fixing devices, overlap, area covered, etc.), making comparisons precarious at best.

In the literature, the effectiveness of the method is assessed differently depending on the authors and the few scientific studies available reported mixed results.

In our questionnaire, tarping effectiveness was measured through two criteria: the presence of knotweed regrowth after tarping, and an evaluation of the method carried out by the managers themselves. In the first case, the managers were asked whether regrowth had been observed during the tarping period and if so, where (within the tarped area, in its immediate vicinity, and/or in the surrounding area). Seventy-eight percent of the respondents reported regrowth within and/or around the tarped area during the first year after installation. However, it would be premature to deduce the ineffectiveness of the method merely from this information. The data collected did not account for the quantity of regrowth or its trend over time. We know that the rhizomes do not die even with three years of tarping [49], so it is unsurprising that regrowth will persist during the first years. This regrowth does not necessarily invalidate the long-term effectiveness of the tarping operation: regular monitoring, uprooting knotweed regrowth, and repairing the tarp if necessary can still lead to eradication of the knotweed stand. In the second case, the answer rate for the evaluations of the method was low because only managers whose operation had ended and whose tarps had been removed were asked to answer this question. This choice was partly based on literature recommendations and on the idea that the effectiveness of a control operation cannot be properly evaluated when the said operation is still ongoing. However, it turned out that most managers (73%) had not yet removed their tarps or even do not intend to remove them, making their tarping operations, in our sense, endless. Consequently, only 22 respondents gave an evaluation of the method, and their evaluations were, as expected, highly contrasted. If the mean score at the removal was 5.7/10 (+/− 3.87 standard deviation), six managers out of 22 reported the eradication of targeted knotweed stands. Although infrequent, such results indicate that tarping can, in some instances, be objectively “effective”. Research efforts should next focus on increasing the number of evaluated operations and elucidating which implementations (i.e., set-ups) or contexts explain the success or failure of tarping operations. Effectiveness assessments should also be based on more quantitative criteria (e.g., density of stem regrowth per square meter) than a simple evaluation by the managers themselves, which remains very subjective and could be influenced by the effort made, the cost of the method, and the manager’s perception and knowledge of knotweed’s biology [19]. Ideally, rhizomes should also be sampled prior to the removal of the tarp in order to assess their vitality and regenerative capacity.

Prolonged knotweed rhizome dormancy is frequently mentioned as a limiting factor to tarping’s effectiveness [31,48,51,52,62,69,77,80,81,82,83], yet we were unable to find any scientific basis for that statement. Prolonged dormancy (also known as “vegetative dormancy”) is a stage in which mature plants remain belowground during one or more growing seasons instead of emerging to grow and acquire resources [84]. This strategy can be a response to a stressful above-ground environment caused by drought, herbivory, etc., [85,86] and/or a consequence of a lack of stored resources [87,88]. Despite the fact that it is a relatively common phenomenon, prolonged dormancy has never been reported in scientific studies on knotweeds. The reference to knotweed rhizome dormancy seems to have originated in “The Knotweed Code of Practice”, published in 2006 by the Environment Agency [62]. In this publication, which itself refers to “unconfirmed observations”, it is stated that the repeated use of herbicides could induce a rhizome dormancy lasting for up to 20 years. Tarping was therefore not originally linked to such observations. Still, by preventing photosynthesis and depriving the plant of water (if an impermeable geomembrane is used), tarping could certainly create the stressful conditions required to induce prolonged dormancy. However, to date, none of the 81 respondents to our questionnaire have reported such a phenomenon and, although it cannot be totally excluded, prolonged dormancy cannot be considered as a limiting factor for tarping’s effectiveness on knotweed.

## 4. Recommendations

The results from our review and questionnaire revealed a great heterogeneity among knotweed tarping practices (e.g., in terms of context, duration, covered area, chosen materials), sometimes reflecting insufficient knowledge of knotweed’s biology and ecology (in particular, on clonal integration and on the expansion capacities of rhizomes). Such variability explains, to a large extent, the difficulty to find comparable operations and the mixed results reported in the literature. Nonetheless, the synthesis presented in this article enable us to propose some practical recommendations to managers.

### 4.1. Cover the Entire Stand

In a number of operations, not all of the knotweed stand was covered. Yet, since knotweeds share resources through clonal integration [26,89], the uncovered part of the plant may compensate for the lack of photosynthesis within the tarped part, as was shown for partial mowing [37]. Therefore, we feel that it is particularly important to implement a tarping operation only when covering the entire stand is feasible, except perhaps in the case (mentioned by a few respondents) where the aim is not to eradicate the knotweed population but to prevent its growth in a specific area (e.g., when the stand impedes visibility or access).

### 4.2. Extend up to 2.5 m

Although doubts remain on the horizontal distance over which knotweed rhizomes can grow, we know that the rhizomatous system can extend several meters beyond the edge of the visible stand [30,41]. Yet, 55% of the respondents only covered the ground 1 m beyond the edge of the stands. According to the latest study by Fennell et al. [41], covering at least 2.5 m, and preferably up to 4 m, beyond the edge of the stand is recommended. Similarly, while rhizomes are found mostly in the first 50 cm of the soil, they can reach a depth 3 m depending on the type of soil, the site characteristics, and the presence of obstacles to circumvent [41]. A quarter of the respondents tried to create an anti-rhizome barrier by burying the edge of the tarp in a trench, but the average depth of this trench was only 58 cm (with trench depth ranging from 10 cm to 2 m). Given the depth sometimes reached by knotweed rhizomes, it may be necessary to install tarps deeper in the soil to achieve a more efficient vertical barrier effect, especially if the distance covered beyond the stand is small.

### 4.3. Maintain the Tarping for at Least Six Years

The recommended tarping duration has steadily increased over time, reflecting a lack of hindsight and empirical development of the method. While it is now quite certain that a short tarping duration cannot kill knotweed rhizomes [49], the most appropriate tarping duration is not yet known. Experiments carried out in the USA over a period of five to eight years have given mixed results [56,61]. The required duration could also depend on the context, the initial vigor of the patch, and its age (in relation to the amount of biomass it has stored underground) [45]. Based on current knowledge, we believe that it is essential to maintain the tarping over at least six years. However, such a long duration is not necessarily adapted to all managers’ objectives and means (financial and human).

### 4.4. Use a Durable Synthetic Tarp

The choice of the fabric should also reflect rhizome lifespan and the duration of the tarping project. We feel it is essential to choose a tarp for its durability, whether it is an agricultural tarp, a geomembrane, or a geotextile. In this respect, we question the relevance of using biodegradable tarps. Their lifespan does not seem sufficient for a successful tarping operation. Therefore, despite the potential environmental impact of synthetic tarps, this choice seems to be the most relevant to truly impede knotweed populations.

### 4.5. Ensure Long-Term Management

Though tarping appears to be a passive technique [46], it is not. Regular monitoring must be carried out to find and repair possible degradations, regrowth must be crushed down under the tarp to prevent it from lifting and piercing the fabric, and sprouts that may have grown through the fabric or in its immediate vicinity must be uprooted. Since most observed deteriorations of the tarps occurred in the year following their installation, regular monitoring makes it possible to detect and fix most problems in due time.

Tarping also requires costly work to remove the tarp at the end of the operation. We found an important discrepancy between theory and practice regarding tarp removal. While guidelines advise removing tarps after a certain period of time, 42% of respondents left the tarp in place at the end of the treatment, or intend to do so. In practice, tarp removal can be difficult because of tarp deterioration (too much fragmentation), the presence of planted vegetation, layers of soil, or simply because of the cost of the operation.

In the literature, it is strongly recommended to revegetate the site after tarp removal [50,51,58,65] since tarping is a non-selective method that makes bare soil prone to invasion by other IAS. Although competition and allelopathy can limit knotweed development [90,91], we recommend revegetation only after tarp removal (unless the aim is to control rather than eradicate the knotweed) as the holes made in the tarp for plantations enable the regrowth of knotweed stems and may limit the effectiveness of the tarping. Though revegetation of the site is obligatory at some point, tarping can be the first step in a more global restoration strategy, implying a long-term investment.

## 5. Conclusions

Through a review and the feedback collected via a questionnaire, we have shown that the available literature on tarping is mainly grey and that there is a great heterogeneity of practices in the field. This reflects the empirical development of the method and demonstrates the need to improve knowledge on tarping as a method of control for Asian knotweed populations.

For the first time, our article synthesized the scattered and often incomplete information available from the literature and compared it with field practices. This important work of descriptive analysis, by making the first state of the art of the tarping, was an essential prerequisite to improving the method.

Although some tarping operations have been shown to successfully eradicate Asian knotweed in small stands, the bibliography and the results of the survey were insufficient to demonstrate the effectiveness of tarping. More data and further analyses, as well as research and experimentation on the functioning (in particular, the life span) of rhizomes, would be useful. The question of the long-term environmental impact of tarping also deserves further consideration: while it is sometimes presented as an alternative to herbicides, the tarping method, especially with synthetic tarps, is not without consequences for the environment.

However, our article highlighted some important aspects to consider to obtain favorable results and proposed practical recommendations for managers to improve the method. Even though tarping does not seem to be a one-size-fits-all solution to eradicate knotweed, it is still a useful control method once knotweed has become a critical management issue.

## Figures and Tables

**Table 1:** Summary of recommendations for the implementation of tarping and comments on its effectiveness. Here we selected the documents from our bibliographical review that we considered to be the most detailed on the tarping method.

Author	Duration of the Tarping	Size of the Stand to Control	Preparatory Work	Timing	Distance beyond the Stand Edge	Type of Tarp	Monitoring	Removal of the Tarp	Tarping Effectiveness
Soll, 2004	Throughout the growing season	Isolated and smaller patches on open terrain	Cutting (possibly followed by tilling)	Right at the beginning of the year or after cutting the plant a couple of times in the spring	At least 2 m (and preferably 7 m)	Thick black plastic or multiple layers of cardboard	At least for 2 years	n/a	No reliable reports of successful knotweed control with covering
McHugh, 2006	Throughout the growing season	Isolated and smaller patches on open terrain	Cutting (possibly followed by tilling)	Right at the beginning of the year or after cutting the plant a couple of times in the spring	At least 2 m (and preferably 7 m)	Thick black plastic or multiple layers of cardboard	Diligent monitoring	n/a	Promising results. Key to success seems to be the strength and the longevity of the geotextile fabrics. Method to consider where herbicide use is restricted
Hallworth and Sellentin, 2011	Several years	Small knotweed outbreaks (<0.001 hectares and <10 stems per m^2^), small patches (e.g., 300 stems or less)	Cutting	n/a	2 up to 10 m	Woven, heavy grade geotextile or thick cardboard + heavy duty black plasti/poly tarp or geotextile fabric	Continously monitoring for several years	n/a	Not effective along stream banks
Anderson, 2012	More than one growing season (up to 3)	Small to medium (0.1 to 2 ha), dense infestations (more than 1000 plants or 30–100% cover)	Cutting	Late spring	Further out	Dark coloured tarp or heavy material (weed barriers or blue poly tarps)	Monitoring for regrowth, rips and tears in the tarp	n/a	This method may need to be used in conjunction with another method to ensure the entire patch is controlled
Godmaire and Houbart, 2014	At least 5 years	Medium (from 10 to 40 m^2^) to large (up to 40 m^2^)	Mowing or cutting	Late spring	A few meters	Dark coloured tarp (as geotextile, geomembrane or polyethylene tarp)	Regular monitoring	n/a	Not recommended in shaded areas
King County, 2015	At least 5 growing seasons (longer if soil is wet)	Reasonably small (50 stems or less), isolated patches	Cutting	At the beginning of the year or after cutting the plant down several times during the growing season	At least 2 m beyond	Geotextile or heavy duty black plastic	Intensive monitoring: every 2 to 4 weeks during the growing season	n/a	Moderately effective
Branquart et al., 2018	More than 5 years, up to 8 years	Small (less than 50 m^2^, because of the high cost of the tarp)	Uprooting or herbicide injection or mowing	Winter or spring (to limit the biomass to be evacuated)	4 to 5 m	Large non-woven geotextile or tarp class 5 (tensile strength greater than 16 kN and density greater than or equal to 240 g/m^2^)	Monitoring at least twice a year	Yes for exposed tarps; No for covered tarps	Mitigation method: very rapid weakening of the knotweed, may lead to its long-term elimination
Comité ZIP des Seigneuries et al., 2018	At least 8 years	n/a	Cutting and excavation (manually or with an excavator) up to 2 m beyond the stand	n/a	At least 2 m	Geomembrane: Texel 800 series or 45 mil ethylene-propylene-diene monomer (EPDM) membrane or Georoute 9	Regular monitoring over several years	Yes	n/a
Cygan, 2018	5 years	n/a	Cutting and spreading a layer of mulch, grass clippings or other material over the cut stems (to prevent them from puncturing the tarp)	June (when rhizomes are weakest)	A few feet beyond	7-mil Black plastic or non-woven geotextile or heavy-duty dark colored tarp	n/a	Yes	Very effective alternative if you wish to avoid the use of herbicides/very successful in sensitive areas
Guerin and Hedont, 2019	Several years, at least 10 years for some	Small (from a dozen to a few hundred m^2^, as it is heavy to implement)	Uprooting or mowing	All seasons	1 to 2 m	New, black and opaque tarps. Geomembrane, agricultural tarp or biodegradable geotextile (non-woven)	Monitoring once or twice a year	Yes	One of the most appropriate methods for eradicating the outbreak of vegetatively propagated herbaceous plants
Lavoie, 2019	Up to 7 years	Tarps are too expensive to be laid out over large areas	n/a	n/a	n/a	Geomembrane or rugged geotextile	Regular monitoring	Yes	Popular but few efficacy tests. Mixed results
SPIGEST, 2019	At least 3 years	Small (less than 500 m^2^)	Mowing in the winter before laying the tarp	Before vegetation regrowth	1 m	Black agricultural tarp (2 layers)	Regular monitoring: at least in the first years, once a month, from May to October.	Yes	Effective method for weakening rhizomial reserves before the establishment of plant competition

## Data Availability

Not applicable.

## Supplementary Materials

The following are available online at https://www.mdpi.com/article/10.3390/plants10102152/s1, Supplementary File S1: Survey on the management of knotweeds through tarping, Supplementary File S2: List of references resulting from the bibliographical research, Supplementary File S3: List of tarping operations identified through the questionnaire and their main characteristics.
