# Supplementary material for: Review of Existing Knowledge and Practices of Tarping for the Control of Invasive Knotweeds
Source: Plants (Basel). 2021 Oct 11;10(10):2152. doi: 10.3390/plants10102152 (PMC8539117; doi:10.3390/plants10102152)
Supplement: Supplementary file 1 [file plants-10-02152-s001.zip › Supplementary 1.pdf]

Supplementary 1. Survey on the management of knotweeds through tarping

A- Respondent identity

Last name:
First name:
Contact details:
Profession:
Employing organization:
Was your employer the tarping project initiator?

- o Yes
- o No
- o I don't know

If not, who was the project initiator?

B -Regarding the tarping project:

B1. Where did the tarping took place? (Country/State/City)

B2. What reason(s) led to choose covering to control knotweeds (several possible answers)?

- □ Previous successful experience(s)
- □ Peer recommendation
- □ Bibliographic search on knotweed control methods
- □ Because of technical or regulation constraints (site accessibility, phytochemical restrictions, etc.)
- □ For the sake of comparison with other methods
- □ As a last resort
- □ I don't know
- □ Other:

B3. What were the targeted objectives (several possible answers)?

- □ To eradicate the knotweed patch
- □ To limit knotweed's spatial expansion on this area
- □ To reduce the knotweed patch's vigour (height, stem density or biomass etc.)
- □ To limit knotweed's dispersal to other areas
- □ I don't know
- □ Other:

B4. What was the initially planned duration of the tarping (in years)?

B5. What determined the planned tarping duration (several possible answers)?

- □ Bibliographic source(s)
- □ Peer recommendations or feedbacks
- □ Personal experience
- □ Alignment with the funding duration of the project
- □ I don't know
- □ Other:

C- Covering area description

C1. What was the surface area of the targeted knotweed patch? (Please specify the unit of measurement (square metres or square feet))

C2. According to you, how old was the knotweed patch at the time of the tarping operation?

- Less than one year
- Between 1 and 3 years
- Between 3 and 10 years
- More than 10 years
- I don't know
- Other:

C3.1. Was the area to be tarped flat?

	0	1	2	3	4	5	6	7	8	9	10	
Totally flat												Very steep (> 50°)

C3.2. Was the area easily accessible?

	0	1	2	3	4	5	6	7	8	9	10	
Very easy to access												Very difficult to access

C3.3. Was the area sunny?

	0	1	2	3	4	5	6	7	8	9	10	
Always in full sun												Always shaded

C3.4. Was the area to be tarped and its immediate surroundings wooded (covered by high shrubs or adult trees)?

	0	1	2	3	4	5	6	7	8	9	10	
Not wooded at all												Very wooded

C4.1. Was the area floor even?

	0	1	2	3	4	5	6	7	8	9	10	
Perfectly even												Totally uneven

C4.2. How was the area soil texture?

	0	1	2	3	4	5	6	7	8	9	10	
Very fine												Very coarse (rocks, fill materials...)

C5. Was the area subjected to floods?

	0	1	2	3	4	5	6	7	8	9	10	
Never												At least once a year

C6. What was the area environment (several possible answers)?

- ☐ Roadside
- ☐ Railway side
- ☐ Riverbank
- ☐ Industrial wasteland, activities area, urban wasteland
- ☐ Park, garden
- ☐ Woodland
- ☐ Agricultural land

- ◻ I don't know
- ◻ Other:

D- Tarping preparation

D1. Has the knotweed patch been controlled just before tarping started (was it mowed, for instance)?
(We would like to know if you prepared the area in order to set up the fabric (e.g. by removing rocks, mowing knotweed stems, or flattening the ground). We do not want to know if this particular knotweed patch has ever been managed.)

- o Yes
- o No
- o I don't know

➢ If the area was prepared before tarping,

D1.1. Which method(s) was used to control the knotweed patch (several possible answers)?

- ◻ Mowing
- ◻ Crushing of rhizomes
- ◻ Manual excavation of rhizomes
- ◻ Mechanical excavation of rhizomes
- ◻ Smoothering with excavator
- ◻ Rocks disposal
- ◻ I don't know
- ◻ Other:

D1.2. When was the patch controlled (in order to cover it with the fabric)?
Please, specify the year and month (or season)

D1.3. How was this time interval chosen (several possible answers)?

- ◻ Organizational constraints (people/material availability)
- ◻ Meteorological conditions
- ◻ Knotweed's biological cycle: before or at the beginning of knotweed emergence in order to limit the amount of wastes too be treated
- ◻ Knotweed's biological cycle: at the end of the growth period (during summer) when rhizomes have the lowest reserves
- ◻ Knotweed's biological cycle: before flowering in order to limit seed production
- ◻ I don't know
- ◻ Other:

D3. According to you, how important this control operation is for the tarping success?

	0	1	2	3	4	5	6	7	8	9	10	
Useless	○	○	○	○	○	○	○	○	○	○	○	Essential

E- Tarping operations

E1. When did the tarping started (the actual operation of covering the plants)?
Please, specify the year and month (or season)

E2. What kind of fabric was used to “tarp” the knotweed patch?

- o A geomembrane (waterproof fabric)
- o A geotextile (permeable fabric)
- o An agricultural tarpaulin
- o I don't know
- o Other:

➢ If you used geotextile

E2.1. Was this geotextile (several possible answers)?

- □ Woven
- □ Non-woven
- □ Synthetic
- □ Biodegradable
- □ In PLA (polylactic acid)
- □ I don't know
- □ Other:

E- Tarping operations (2)

E3. Can you specify the brand, model, material, weight and colour of the “fabric” (geotextile, geomembrane, tarp, etc.)?

E4. Has the entirety of the knotweed patch been covered?

- o Yes
- o No
- o I don't know

E4.1. If not, why?

E5. How far from the knotweed patch’s edge has the fabric been installed (distance between the patch’s edge and the fabric’s edge, in metres)?
(If you use another unit of measurement, please specify)

E6. Did you use several fabric strips?

- o Yes
- o No
- o I don't know

➢ Strips Overlap

E6.1. If you used several strips of fabric, how did you join them?

- □ Glue
- □ Tape
- □ Heat sealing
- □ Clips
- □ I don't know
- □ Other:

E6.1.1. If you used glue, what kind of glue was it?

E6.1.2. If you used tape, what kind of tape was it?

E6.1.3. If you used clips, what was the spacing between each clip (in centimetres)?
(If you use another unit of measurement, please specify)

E6.2. What overlap did you choose to join the fabric strips (in centimetres)?
(If you use another unit of measurement, please specify)

E- Tarping operations (3)

E7. Did you use clips to fix the fabric to the ground?
- o Yes
- o No
- o I don't know

➢ If you used clips

E7.1. What kind of clips did you use?
- o Concrete reinforcing bar
- o Metal clips
- o Wooden clips
- o Plastic clips
- o Biodegradable clips
- o I don't know
- o Other:

E7.2. What was the spacing between each clip (in centimetres)?
(If you use another unit of measurement, please specify)

E- Tarping operations (4)

E8. How did you fix or weigh down the fabric (in addition to clips if you used them)?
- □ Heavy objects (rocks, logs, sand bags…)
- □ Soil, sand, or gravel covering
- □ I don't know
- □ Other:

E8.1. If you covered the fabric, can you specify the layer thickness (in centimetres)?
(If you use another unit of measurement, please specify)

E9. Did you dig a trench around the tarping area?
- o Yes
- o No
- o I don't know

➢ If you dug a trench

E9.1. How deep was the trench dug (in centimetres)?

(If you use another unit of measurement, please specify)

E9.2. What was the purpose of that trench (several possible answers)?

- □ To fix the fabric edges
- □ To create a vertical rhizome barrier with the fabric edges
- □ To create a vertical rhizome barrier with another fabric or material (planks etc.)
- □ I don't know
- □ Other:

E9.3. If you used another fabric to create a rhizome barrier, can you specify the brand and model of that fabric?

E- Tarping operations (5)

E10. Were there any obstacles on the tarped area (trees, rocks, etc.)?

	0	1	2	3	4	5	6	7	8	9	10	
None	◯	◯	◯	◯	◯	◯	◯	◯	◯	◯	◯	Many obstacles

E10.1. If there were obstacles, how did you adapt to them?

E11. Has the fabric unwillingly been torn up during installation?

	0	1	2	3	4	5	6	7	8	9	10	
No	◯	◯	◯	◯	◯	◯	◯	◯	◯	◯	◯	Many holes

E11.1. If yes, by what?

- □ Rocks, rubbles, rebars, branches…
- □ Mowed stem residues
- □ Machine trampling
- □ I don't know
- □ Other:

E12. Who laid down (installed) the fabric?

- □ A private company
- □ Myself
- □ An association
- □ I don't know
- □ Other:

E12.1. Can you specify its name?

E13. Have plants been planted within the tarped area?

- o Yes
- o No
- o I don't know

➢ If you planted plants

E13.1. What did you use?

- ◻ Seedlings
- ◻ Cuttings
- ◻ Plantations
- ◻ I don't know
- ◻ Other:

E13.2. How many months after tarping were these plants planted?

E13.3. B2. What reason(s) led to this planting (several possible answers)?

- ◻ Competition between plants and knotweeds.
- ◻ Revegetation with native species
- ◻ Avoid erosion
- ◻ Aestheticism
- ◻ I don't know
- ◻ Other:

E- Tarping operations (6)

E14. According to you, how difficult is the fabric (tarp) installation?

	0	1	2	3	4	5	6	7	8	9	10	
Very easy	◯	◯	◯	◯	◯	◯	◯	◯	◯	◯	◯	Very hard

E15. According to you, how important this step is for the success of the project?

	0	1	2	3	4	5	6	7	8	9	10	
Useless	◯	◯	◯	◯	◯	◯	◯	◯	◯	◯	◯	Essential

F - Monitoring during operations

F1- After it was covered, has the area been monitored?

- o Yes
- o No
- o I don't know

➢ Follow-up during covering

F1.1. How frequently were follow-up investigations conducted?

- o Once a year
- o Twice a year
- o Thrice a year
- o I don't know
- o Other:

F1.2. What kind of follow-up investigation was performed?

- ◻ Fabric fixing
- ◻ Knotweed uprooting

- ◻ Mowing (in or around the area)
- ◻ Stem crushing under the fabric
- ◻ Replacement of dried or ill vegetation
- ◻ I don't know
- ◻ Other:

F1.3. Who conducted those follow-up investigations?
- ◻ A private company
- ◻ Myself
- ◻ An association
- ◻ I don't know
- ◻ Other:

F1.3.1. Can you specify its name?

F- Monitoring during operations (2)

F2. During the time the fabric (tarp) was in place, did you observe any knotweed regrowth?
- o Yes
- o No
- o I don't know

- ➢ If there were knotweed regrowth

F2.1. Where did knotweed regrew (several possible answers)?
- ◻ At the level of clips
- ◻ At the level of overlapping strips
- ◻ At the junction with obstacles
- ◻ Within holes in the fabric
- ◻ At the level of planted vegetation
- ◻ In the immediate vicinity of the tarped area (< 2 metres)
- ◻ In the surrounding of the tarped area (> 2 metres)
- ◻ I don't know
- ◻ Other:

F2.2. How many months after the area was tarped was the first knotweed regrowth observed?

F- Monitoring during operations (3)

F3. Did you notice any deteriorations of the tarped setting (fabric, junctions, plants, etc.)?
- o Yes
- o No
- o I don't know

- ➢ If there were deteriorations

F3.1. After how long did you notice those deteriorations (in months)?

F3.2. Was it about the fabric itself?
- o Yes

- No
- I don't know
- Other:

F3.2.1. If so, what were the reasons for those fabric deteriorations (several possible answers)?

- ☐ Fabric durability
- ☐ Trampling
- ☐ Vandalism
- ☐ Meteorological conditions: wind
- ☐ Meteorological conditions: flood(s)
- ☐ Meteorological conditions: ice
- ☐ I don't know
- ☐ Other:

F3.2. Was it about the fabric fixation?

- Yes
- No
- I don't know

F3.2.1. If so, what were the reasons for those fixation deteriorations (several possible answers)?

- ☐ Vandalism
- ☐ Trampling
- ☐ Inadequate weights
- ☐ Meteorological conditions: wind
- ☐ Meteorological conditions: flood(s)
- ☐ Meteorological conditions: ice
- ☐ Fixation durability
- ☐ I don't know
- ☐ Other:

F- Monitoring during operations (4)

F4. Did you interrupted the tarping project earlier than the initially planned date (project abandonment)?

- Yes
- No
- I don't know

➢ If you stopped covering before the initial term

F4.1. How long after installation did you removed the fabric?

F4.2. Why did you removed it?

- ☐ Whole fabric deterioration
- ☐ Fabric tearing
- ☐ Lack of maintenance
- ☐ Lack of funding
- ☐ Discouragement
- ☐ Project abandonment

- ☐ I don't know
- ☐ Other:

F- Monitoring during operations (5)

F5. According to you, are follow-up investigations important during a tarping operation?

	0	1	2	3	4	5	6	7	8	9	10	
Useless	◯	◯	◯	◯	◯	◯	◯	◯	◯	◯	◯	Essential

G- Tarp/Geotextile/Geomembrane removal

G1. Did you remove the fabric yet?

- o Yes
- o No, because the planned tarping duration has not been reached yet.
- o No, because the fabric is totally deteriorated
- o No, because we do not intend to move it
- o I don't know
- o Other:

➢ If you removed the fabric

.

G2. How long after installation did you remove the fabric?

G3. At the time of removal, what was the fabric condition?

- o Excellent (no holes)
- o Good condition (few holes)
- o Bad condition (numerous holes)
- o Totally deteriorated
- o I don't know
- o Other:

G4. At the time of removal, did you consider that the knotweed patch was eradicated?

	0	1	2	3	4	5	6	7	8	9	10	
Yes, totally	◯	◯	◯	◯	◯	◯	◯	◯	◯	◯	◯	No, not at all

G5. At the time of removal, where did you see knotweed regrowth?

- ☐ Within the tarped area
- ☐ In the immediate vicinity of the tarped area (< 2 metres)
- ☐ In the surrounding of the tarped area (> 2 metres)
- ☐ I don't know
- ☐ Other:

G5.1. If there were knotweed sprouts in the tarped area, how would you describe this knotweed cover compared to the cover initially tarped?

	0	1	2	3	4	5	6	7	8	9	10	
Much less extended	◯	◯	◯	◯	◯	◯	◯	◯	◯	◯	◯	Much more extended

G5.2. If there were knotweed sprouts in the tarped area, how would you describe the knotweed patch's vigour compared to its initial vigour?

	0	1	2	3	4	5	6	7	8	9	10	
Less vigorous	○	○	○	○	○	○	○	○	○	○	○	More vigorous

G6. At the time of the fabric removal, how did you judge the method's effectiveness?

	0	1	2	3	4	5	6	7	8	9	10	
Ineffective	○	○	○	○	○	○	○	○	○	○	○	Very effective

H- Post-control observations

H1. After the fabric removal, has the area been managed?

- o Yes
- o No
- o I don't know
- o Other:

➢ If the area was managed

H1.1. What kind of management was performed (several possible answers)?

- □ Seeding
- □ Cutting
- □ Planting
- □ Mowing
- □ Uprooting
- □ I don't know
- □ Other:

H1.2. If so, how many months after the fabric removal?

H- Post-control observations (2)

H2. At the time of your latest observation of the area (specify below), did you see knotweed regrowth?

- □ Yes, in the formerly tarped area
- □ Yes, in the immediate vicinity of the formerly tarped area (< 2 metres)
- □ Yes, in the surrounding of the formerly tarped area (> 2 metres)
- □ Yes, but I don't remember exactly where
- □ No
- □ I don't know
- □ Other:

H2.1. When was your last observation of the area made? *Please use the European date format (DD/MM/YYYY)*

H3. At the time of your latest area observation, how did you judge the method's effectiveness?

	0	1	2	3	4	5	6	7	8	9	10	
Ineffective	◯	◯	◯	◯	◯	◯	◯	◯	◯	◯	◯	Very effective

I- Cost

I1. Could you give us an estimation of the total cost of the tarping operation (including the cost of prior operations, of the fabric, plants, follow-up investigations, fabric removal, etc.)?

I2. Compared to other knotweed control methods, do you consider tarping as:

	0	1	2	3	4	5	6	7	8	9	10	
Very cheap	◯	◯	◯	◯	◯	◯	◯	◯	◯	◯	◯	Very expensive

I3. Compared to other knotweed control methods, do you consider tarping as:

	0	1	2	3	4	5	6	7	8	9	10	
Time sparing	◯	◯	◯	◯	◯	◯	◯	◯	◯	◯	◯	Very time-consuming

J- Manager's perception

J1. According to you, what is your level of expertise on knotweeds?

	0	1	2	3	4	5	6	7	8	9	10	
Novice	◯	◯	◯	◯	◯	◯	◯	◯	◯	◯	◯	Expert

J2. Would you like to use tarping on other areas?

- Yes
- No
- I don't know
- Other:

J2.1. If so, will you change or improve the method? How?

J4. Do you have any general comments on tarping?

J5. Can you suggest any other people or organizations we should contact regarding management by tarping?

J6. General comments:
