# Supplementary material for: Review of Existing Knowledge and Practices of Tarping for the Control of Invasive Knotweeds
Source: Plants (Basel). 2021 Oct 11;10(10):2152. doi: 10.3390/plants10102152 (PMC8539117; doi:10.3390/plants10102152)
Supplement: Supplementary file 1 [file plants-10-02152-s001.zip › Supplementary 2.pdf]

Supplementary 2. List of references resulting from the bibliographical research

1. Anderson, H., 2012. Invasive Japanese Knotweed (Fallopia japonica (Houtt.) Best Management Practices in Ontario. Ontario Invasive Plant Council, Peterborough (Canada).
2. Association des Gestionnaires d'Espaces Naturels Bretons, 2017. Journée d'échanges sur la gestion des EEE végétales terrestres 7 novembre 2017/Landes de Ploumanac'h, Compte rendu, Perros-Guirec.
3. Association ECHEL, 2018. Régulation des renouées asiatiques (Fallopia japonica, Fallopia sachalinensis, Fallopia x bohemica). Fiche technique N° 1, Nans sous Sainte Anne.
4. Association Rivière Rhône Alpes (ARRA), 2011. Renouées du Japon: gestion et lutte. Actes des journées techniques. Association Rivière Rhône Alpes (ARRA), Grenoble.
5. Boileau, N., Griggs, R., 2018. Douze années de gestion de la Renouée asiatique (Reynoutria japonica) sur le bassin versant du Vicoin (2007-2018). Bulletin Scientifique BIOTOPES 53 n°35, 7–16.
6. Branquart, E., Trojan, M., Vanparys, V., 2018a. La gestion des renouées asiatiques. Technique d'atténuation. Gestion par bâchage. Service Public de Wallonie, Cellule interdépartementale Espèces invasives, (Belgium).
7. Branquart, E., Trojan, M., Vanparys, V., 2018b. Les renouées asiatiques en Wallonie: 10 techniques pour mieux les combattre. Service Public de Wallonie, Cellule interdépartementale Espèces invasives, (Belgium).
8. Clements, D.R., Larsen, T., Grenz, J., 2016. Knotweed management strategies in North America with the advent of widespread hybrid Bohemian knotweed, regional differences, and the potential for biocontrol via the psyllid Aphalara itadori Shinji. Invasive Plant Science and Management 9, 60–70. https://doi.org/10.1614/IPSM-D-15-00047.1
9. Coeur Emeraude, 2013. Lutte contre la renouée du Japon, espèce invasive. Réunion d'information organisée par COEUR Emeraude le 1er juillet 2013 à St Lunaire, dans le cadre de la mise en place du futur PNR, St Lunaire.
10. Comité ZIP des Seigneuries, Comité ZIP Jacques Cartier, Conseil québécois des espèces exotiques envahissantes, 2018. Renouée du Japon Protocole de contrôle, Montréal (Canada).
11. Conseil québécois des espèces exotiques envahissantes, Contrôle de la renouée du Japon http://cqeee.org/?page_id=622 (accessed 31 July 2020).
12. Contrat de rivière de la Lanterne, 2011. Contrat de rivière de la Lanterne - Bilan d'étape, Vesoul.
13. Contrat de rivière Romanche, SACO, 2016. Travaux de lutte contre les plantes invasives sur le bassin de la romanche - Phase 2, Bourg d'Oisans.
14. Cottet, M., Rivière-Honegger, A., Vaudor, L., Colombain, L., Dommanget, F., Evette, A., 2020. The end of a myth: Solving the knotweeds invasion "problem." Anthropocene 30, 100240. https://doi.org/10.1016/j.ancene.2020.100240
15. Cygan, D., 2018. Preventing the Spread of Japanese knotweed (Reynoutria japonica) Best Management Practices. New Hampshire Department of Agriculture, Markets & Food.

16. Derickx, L.M., Antunes, P.M., 2013. A Guide to the Identification and Control of Exotic Invasive Species in Ontario's Hardwood Forests, Algoma University. ed. Ontario (Canada).
17. Direction régionale de l'environnement, de l'aménagement et du logement Midi-Pyrénées, 2014. Plan régional d'actions: plantes exotiques envahissantes en Midi-Pyrénées. 2013-2018. Direction régionale de l'environnement, de l'aménagement et du logement Midi-Pyrénées, Toulouse.
18. Eisel, T., 2018. Erfahrungen bei der herbizidfreien Bekämpfung von Staudenknöterich-Beständen. Ing.- Büro für Garten- und Landschaftsplanung, Mühlbeck (Germany).
19. ENSAIA, 2016. Lutte contre des plantes invasives, les Renouées asiatiques. Projet Professionnel 2015-2016, Vandœuvre-lès-Nancy.
20. Environment Agency, 2006. Managing Japanese knotweed on development sites: The Japanese knotweed code of practice. Environment Agency, Bristol (United Kingdom).
21. EPL Tournus, Lycée de l'Horticulture et du Paysage, 2017. Un projet d'expérimentation de lutte contre la Renouée du Japon à l'initiative des élèves du Lycée de l'Horticulture et du Paysage de Tournus, Tournus.
22. Evette, A., Breton, V., Petit, A., Dechaume-Moncharmont, C., Brasier, W., 2019. Les techniques de bâchage pour le contrôle de la renouée. Ingénieries 62–67. https://doi.org/10.14758/SET-REVUE.2019.1.11
23. FRAPNA, 2011. Espèces exotiques envahissantes plantes invasives. bilan 2011. FRAPNA Haute Savoie.
24. Gerber, E, Murrell, C., Krebs, C., Bilat, J., Schaffner, U., 2010. Evaluating non-chemical management methods against invasive exotic knotweeds, Fallopia spp. CABI, Egham (United Kingdom).
25. Godmaire, H., Houbart, C., 2014. Renouée du Japon Guide technique de contrôle mécanique. Ville de Granby Conseil québécois des espèces exotiques envahissantes – CQEEE Fondation pour la sauvegarde des écosystèmes du territoire de la Haute-Yamaska - Fondation SÉTHY, Granby (Canada).
26. Guerin, M., Hedont, M., 2019. Plantes envahissantes : Pratiques des gestionnaires d'espaces verts. Recueil d'expériences. Plante & Cité, Angers.
27. Hallworth, J., Sellentin, E., 2011. Understanding and Controlling Invasive Knotweeds in BC. (Canada).
28. Haury, J., Hudin, S., Matrat, R., Anras, L., et al., 2010. Manuel de gestion des plantes exotiques envahissant les milieux aquatiques et les berges du bassin Loire-Bretagne. Fédération des conservatoires d'espaces naturels.
29. INNSA, 2017. Code of practice. Managing Japanese knotweed. INNSA (Invasive Non-Native Specialists Association), Thornby (United Kingdom).
30. Jones, D., Bruce, G., Fowler, M.S., Law-Cooper, R., Graham, I., Abel, A., Street-Perrott, F.A., Eastwood, D., 2018. Optimising physiochemical control of invasive Japanese knotweed. Biol Invasions 20, 2091–2105. https://doi.org/10.1007/s10530-018-1684-5
31. Kaczmarek-Derda, W., Holm, A.-K., Brandsæter, L., Solhaug, K., Fløistad, I., 2019. Survival time of rhizomes of invasive Reynoutria taxa when above-ground shoot production is prevented by covering with geotextile. Presented at the EMAPi 15 - 15th

Conference on Ecology and Management of Alien Plant invasions, 9-13 september, Prague (Czech Republic).
32. King County, 2016. Shadow Lake Integrated Aquatic Vegetation Management Plan. King County Department of Natural Resources and Parks Water and Land Resources Division Noxious Weed Control Program, Seattle (USA).
33. King County, 2015. Best management practices. Invasive Knotweeds. King County Noxious Weed Control Program, Seattle (USA).
34. King County, 2007. Green River CWMA Invasive Knotweed Control. King County Noxious Weed Control Program Water and Land Resources Division Department of Natural Resources and Parks, Seattle (USA).
35. King County, 2004. Hatchery Natural Area. Site Management Guidelines. King County Department of Natural Resources and Parks, Water and Land Resources Division, Seattle (USA).
36. Larsen, T., 2013. Biology, ecological impacts, and management of Japanese knotweed (Polygonum cuspidatum syn. Fallopia japonica) in Novia Scotia, Halifax (Canada).
37. Lavoie, C., 2019. 50 plantes envahissantes: protéger la nature et l'agriculture. Publications du Québec, Québec (Canada).
38. Le Roux, Y., et al., 2017. Gestion de la renouée par bâchage. Presented at the Colloque SPIGEST, La gestion intégrée des renouées invasives, 4 et 5 octobre 2017, Laxou.
39. Louboutin, B., 2010. Les plantes invasives de Brennilis situation et moyens de lutte, Brennilis.
40. LUPIN, 2014. Lupin se mobilise pour la biodiversité, lutte contre les plantes invasives. LUPIN (Lutte contre les plantes invasives), GDON Flandre Maritime.
41. Mantzou, P., 2008. Japanese knotweed: impact on brownfield development and discussion on newly implemented innovative solutions. Brownfields IV WIT Transactions on Ecology and the Environment 107, 65–75. https://doi.org/10.2495/BF080071
42. McHugh, J.M., 2006. A review of literature and field practices focused on the management and control of invasive knotweed. The Nature Conservancy, West Haven (USA).
43. Miller, J.H., Manning, S.T., Enloe, S.F., 2013. A management guide for invasive plants in southern forests. Gen Tech Rep SRS–131 Asheville NC US Dep. Agric. For. Serv. South. Res. Stn. 120 P 131, 1–120.
44. Mitchell, T., Bartenstein, J., 2011. Managing Japanese Knotweed: Two Small-Scale Strategies. Ecological Landscape Alliance. URL https://www.ecolandscaping.org/09/landscape-challenges/invasive-plants/managing-japanese-knotweed-two-small-scale-strategies/ (31 July 2020).
45. Nickelson, S., 2017. Taylor Townsite Habitat Restoration Project As-Built Document, 2007 – 2017. Watershed Management Division Seattle Public Utilities, Seattle (USA).
46. Nickelson, S., 2014. Webster Creek and Walsh Lake Riparian Habitat Restoration Project As-Built Document. Watershed Management Division Seattle Public Utilities, Seattle (USA).
47. Nickelson, S., 2013. Knotweed Treatment through 2012 Cedar River Municipal Watershed. Annual Report Libraries, Utilities, and Center Committee Seattle City Council. Seattle Public Utilities, Watershed Services Division, Seattle (USA).

48. ONF, 2009. Ruisseaux de têtes de bassins et faune patrimoniale associée, LIFE04NAT/FR/000082. Rapport concernant les actions menées pour la reconstitution de ripisylve Lutte contre une espèce invasive : la Renouée du Japon Travaux de gestion de la ripisylve. Office National des Forêts, Belfort.
49. Parc naturel régional des Ballons des Vosges, 2017. Les plantes invasives du Parc naturel régional des Ballons des Vosges : Connaissance et moyens de lutte. Les renouées asiatiques, Munster.
50. Parkinson, H., Mangold, J., 2010. Biology, ecology and management of the knotweed complex (polygonum spp.). Montana State University Extension, (USA).
51. Pipet, N., 2012. Les renouées asiatiques. Réunion ORENVA –23 mai 2012. IIBSN, Melle.
52. Réseau IDEAL, IDEAL connaissances, 2012. Colloque national Renouées asiatiques. 23 et 24 octobre 2012, St Etienne.
53. Sarat, E., Mazaubert, E., Dutartre, A., Poulet, N., Soubeyran, Y., 2015. Les espèces exotiques envahissantes dans les milieux aquatiques : connaissances pratiques et expériences de gestion (Volume 2 - Expériences de gestion), Comprendre pour agir. Onema.
54. Schmiedel, D., Wilhelm, E.-G., Roth, M., Scheibner, C., Nehring, S., Winter, S., 2016. Evaluation system for management measures of invasive alien species. Biodivers Conserv 25, 357–374. https://doi.org/10.1007/s10531-016-1054-5
55. Soll, J., 2004. Controlling knotweed (Polygonum cuspidatum, P. sachalinense, P. polystachyum and hybrids) in the Pacific Northwest. The Nature Conservancy, Portland (USA).
56. Solviche, A., 2017. La Renouée du Japon. Retour sur 20 ans d'expérience, Nans sous Sainte Anne.
57. Spiegelberger, T., Gerber, E., Schaffner, U., 2006. Reynoutria 2006 - Synthese. Ecologie, impact sur l'environnement et gestion des renouées envahissantes. CABI.
58. SPIGEST, 2019. Guide Pratique. Pour une gestion raisonnée des renouées asiatiques. URL https://spigestinvasives.files.wordpress.com/2019/04/guide-de-gestion-des-renouc3a9es-asiatiques-invasives.pdf (accessed 8.14.20).
59. Ståhlberg, M., 2020. Japanese knotweed: the "nightmare plant" haunting Scania. Lund University, Lund (Norway).
60. Syndicat mixte de la Vallée de l'Orge Aval, 2009. Bilan du programme de lutte contre la Renouée du Japon 2005-2009. Syndicat mixte de la Vallée de l'Orge Aval, Viry-Chatillon.
61. Terrin, E., 2011. Les espèces végétales exotiques envahissantes dans l'Ain : fiches de synthèse par espèce. Conservatoire Botanique National Alpin.
62. UICN France, 2015. Les espèces exotiques envahissantes sur les sites d'entreprises. Livret 2 : Identifier et gérer les principales espèces. Paris.
63. Varray, S., Haury, J., Hudin, S., et al., 2018. Manuel de gestion des espèces exotiques envahissantes du bassin Loire-Bretagne. Fédération des Conservatoires d'espaces naturels.
