# Supplementary material for: Review of Existing Knowledge and Practices of Tarping for the Control of Invasive Knotweeds
Source: Plants (Basel). 2021 Oct 11;10(10):2152. doi: 10.3390/plants10102152 (PMC8539117; doi:10.3390/plants10102152)
Supplement: Supplementary file 1 [file plants-10-02152-s001.zip › Supplementary 3.pdf]

Supplementary 3. List of tarping operations identified through the questionnaire and their main characteristics.

	City and country of the tarping operation	Date of tarp laying	Stand area (m2)	Type of tarp used	Tarp material	Distance from the stand edge covered by the tarp (m)	Tarp removal (duration of tarping)	Evaluation of effectiveness at tarp removal (0 = ineffective / 10 = very effective)
1	Chalon- sur Saône (71), France	2017	2	Geotextile	Synthetic	1	Not yet	n/a
2	Chalon- sur Saône (71), France	2018	1000	Geotextile	Synthetic	2	Not yet	n/a
3	Moux en Morvan (71), France	2012	10	Geotextile	n/a	1	No	n/a
4	Merville (14), France	2015	60	Geomembrane	Synthetic	5	No	n/a
5	Crépol (26), France	2008	15	Agricultural tarpaulin	Synthetic	1	No	n/a
6	? (88), France	2017	10	Agricultural tarpaulin	Synthetic	0,5	Yes (1 year)	2
7	Sens (89), France	2018	3	Other	Synthetic	2	Not yet	n/a
8	Arles sur Tech (66), France	2015	10	Agricultural tarpaulin	Synthetic	1	Yes (2 years)	10
9	Orcival (63), France	2015	100	Other	n/a	0,5	No	n/a
10	Ouessant (29), France	2016	300	Agricultural tarpaulin	Synthetic	1	Not yet	n/a
11	Sequedin (59), France	2015	10	Geotextile	PLA	2	No	n/a
12	La Terrasse sur Dorlay (42), France	2014	300	Agricultural tarpaulin	Synthetic	n/a	Yes (3 years)	10
13	Laguiole (12), France	2013	300	Geomembrane	Synthetic	1	Not yet	n/a
14	Estouy (45), France	2016	300	Agricultural tarpaulin	Synthetic	2	No	n/a
15	Cour Cheverny (41), France	2005	50	Geotextile	Biodegradable	0,5	No	n/a
16	Yffiniac (22), France	2015	35	Geotextile	Biodegradable	0,5	No	n/a
17	Harfleur (76), France	2014	225	Geotextile	Synthetic	0	Yes (1 year)	7
18	Annemasse (74), France	2020	245	Geotextile	n/a	2	Not yet	n/a
19	Rigny-Ussé (37), France	2018	100	Geotextile	Biodegradable	1	Not yet	n/a
20	Combourg (35), France	2009	50	Agricultural tarpaulin	Synthetic	0	No	n/a
21	Douy (28), France	2014	50	Geomembrane	Synthetic	0,5	No	n/a
22	Chateaudun (28), France	2015	30	Geomembrane	n/a	0,3	Yes (2 years)	2
23	Jonchery sur Vesle (51)	n/a	40	Geotextile	n/a	1	Yes (5 years)	10
24	La Bourboule (63)	2006	360	Other	Synthetic	n/a	Yes (2 years)	n/a
25	Vignieu (38)	2020	10	Agricultural tarpaulin	Synthetic	2	Not yet	n/a
26	Ploubazlanec (22)	2020	500	Agricultural tarpaulin + Geotextile	Synthetic	2	No	n/a
27	Cessieu (38), France	2019	450	Geotextile	Synthetic	1	Not yet	n/a
28	St Cosme en Vairais (72), France	2015	20	Agricultural tarpaulin + Geotextile	Synthetic	2	Yes (5 years)	8
29	Lac de Save (38), France	2018	75	Geotextile	n/a	2	Not yet	n/a
30	Crépieux-la-Pape (38)	2019	5000	Geotextile	Synthetic	2	Not yet	n/a
31	Montceau les Mines (71), France	2010	2000	Geotextile	Synthetic	1	No	n/a
32	Jarnosse (42), France	2018	140	Agricultural tarpaulin	Synthetic	2	No	n/a
33	La Chappelle en Valgaudémar (05)	n/a	50	Geomembrane	Synthetic	0,5	Yes (5 years)	10
34	Saint Laurent (31), France	2017	1000	Geotextile	Synthetic	2	No	n/a
35	Fort Mahon Plage (80)	2009	100	Geotextile	Synthetic	1	No	n/a
36	Obernai (67), France	2011	50	Geotextile	Synthetic	5	No	n/a
37	Arlon, Belgique	2017	50	Geomembrane	Synthetic	2	Not yet	n/a
38	Les Noës (42), France	2008	10	Agricultural tarpaulin	Synthetic	2	No	n/a
39	Ernée (53), France	2018	60	Geotextile	Biodegradable	n/a	No	n/a
40	Ugine (73), France	2018	200	Geomembrane	Synthetic	1	Not yet	n/a
41	Velzic (15),France	2018	20	Agricultural tarpaulin	Synthetic	1	Not yet	n/a
42	Chalon sur Saône (71), France	2018	90	Geotextile	Synthetic	0	Not yet	n/a
43	La Bridoire (73), France	2016	20	Geotextile	Synthetic	1	Not yet	n/a
44	Tournus (71), France	2015	150	Geomembrane	Synthetic	0,5	No	n/a
45	Cerfontaine, Belgique	2011	100	Other	Synthetic	0	No	n/a
46	Laguiole (12), France	2014	30	Geomembrane	Synthetic	1	Not yet	n/a
47	Bonson (42), France	2014	40	Agricultural tarpaulin	Synthetic	2	Yes (5 years)	9
48	Lucenay-l'Évêque (71), France	2012	400	Geotextile	Synthetic	1	Not yet	n/a
49	Lacanau (33), France	2013	n/a	Agricultural tarpaulin	Synthetic	1	Not yet	n/a
50	Chantilly (60), France	2020	6	Agricultural tarpaulin	Synthetic	0	Not yet	n/a
51	Le Freney d'Oisans (38), France	2016	30	Geomembrane	Synthetic	1	Not yet	n/a
52	Ornon (38), France	2016	150	Geomembrane	Synthetic	1	Other	n/a
53	Saint Martin sur le Pré (51), France	2014	n/a	Geotextile	n/a	n/a	No	n/a
54	Lochieu (01), France	2017	15	Agricultural tarpaulin	Synthetic	1	Not yet	n/a
55	Port-Brillet (53), France	2012	4	Geotextile	n/a	0	Yes (2 years)	0
56	Banassac-Canilhac (48), France	2016	100	Agricultural tarpaulin	Synthetic	n/a	No	n/a
57	Vandoeuvre-lès-Nancy (54), France	2016	400	Agricultural tarpaulin	Synthetic	1	Yes (?)	n/a
58	Lunac (12), France	2011	55	Agricultural tarpaulin	Synthetic	1	No	n/a
59	Brest (29), France	2015	50	Geomembrane	Synthetic	2	n/a	n/a
60	Grandsaigne (19), France	2016	30	Agricultural tarpaulin	Synthetic	1	No	n/a
61	Taden (22) France	2003	2000	Geotextile	Biodegradable	n/a	No	n/a
62	Maulévrier-Sainte-Gertrude (76), France	2013	50	Geomembrane	Synthetic	1	Yes (5 years)	5
63	Tancarville (76), France	2009	100	Agricultural tarpaulin	Synthetic	1	Yes (2 years)	3
64	Sainte Suzanne (53), France	2012	6	Geomembrane	Synthetic	1	No	n/a
65	Evreux (27), France	2018	600	Agricultural tarpaulin	Synthetic	1	No	n/a
66	Saint Alban (22), France	2019	300	Geotextile	Biodegradable	2	No	n/a
67	Le Havre (76), France	2004	600	Geotextile	Synthetic	n/a	Yes (8 years)	3
68	Quimper (29), France	2005	200	Geomembrane	Synthetic	1	Yes (10 years)	5
69	Oullins (69), France	2015	150	Geomembrane	Synthetic	2	Yes (5 years)	10
70	Brionne (27), France	2018	150	Geotextile	Synthetic	n/a	No	n/a
71	Château Landon (77), France	2017	90	Geotextile	Biodegradable	1,5	No	n/a
72	Chilliwack (BC), Canada	2015	90	Geomembrane	Synthetic	1	No	n/a
73	Fredericton (NB), Canada	2017	200	Agricultural tarpaulin	Synthetic	5	Not yet	n/a
74	Lexington (Masachusetts), USA	2005	232	Agricultural tarpaulin	Synthetic	n/a	Yes (4 years)	5
75	Jessnitz (Saxony-Anhalt), Germany	2005	n/a	Geotextile	Synthetic	1,5	No	n/a
76	(New Hampshire), USA	2008	74	Agricultural tarpaulin + Geotextile	Synthetic	3	Yes (?)	n/a
77	Buffalo (NY), USA	2011	46	Geotextile	Synthetic	1,5	Yes (4 years)	0
78	Granby (QC), Canada	2015	6	Geomembrane	Synthetic	2	Not yet	n/a
79	Bled , Slovenia	2016	350	Geotextile	Synthetic	1	Yes (3 years)	10
80	Watkins Glen (NY), USA	2010	111	Agricultural tarpaulin	Synthetic	1	No	n/a
81	Esbeek (Landgoed De Utrecht), Nederland	2012	100	Geotextile	Synthetic	5	Yes (5 years)	0
